# Microbiome-Driven Bioactives for Chronic Wound Repair: Microbial Metabolites, Host–Microbe Mechanisms and Paths to Clinical Translation

**DOI:** 10.3390/molecules31132229

**Published:** 2026-06-24

**Authors:** Juliana Garcia, Jani Silva, Maria José Alves, Irene Gouvinhas

**Affiliations:** 1 AquaValor, Centro de Valorização e Transferência de Tecnologia da Água, Associação, Rua Dr. Júlio Martins nº 1, 5400-342 Chaves, Portugal; 2 LiveWell, Research Centre for Active Living & Wellbeing, Instituto Politécnico de Bragança, 5300-253 Bragança, Portugal; 3 Centre for the Research and Technology of Agroenvironmental and Biological Sciences, CITAB, Institute for Innovation, Capacity Building and Sustainability of Agri-Food Production, Inov4Agro, Universidade de Trás-os-Montes e Alto Douro, UTAD, Quinta de Prados, 5000-801 Vila Real, Portugal; 4 CIMO, Centro de Investigação de Montanha, Instituto Politécnico de Bragança, 5300-253 Bragança, Portugal; 5 Animal and Veterinary Research Center (CECAV), University of Trás-os-Montes and Alto Douro, 5000-801 Vila Real, Portugal; 6 Associate Laboratory of Animal and Veterinary Sciences (AL4AnimalS), 5000-801 Vila Real, Portugal

**Keywords:** human microbiome, chronic wounds, wound healing, microbial metabolites, microbiome-targeted molecules, probiotics, prebiotics, postbiotics, biofilms

## Abstract

Chronic wounds represent a substantial and growing clinical burden, yet durable healing remains difficult to achieve in a large proportion of patients. The skin microbiome plays a central role in this challenge: in healthy tissue, resident microorganisms support barrier integrity and calibrate immune responses, whereas in chronic wounds, community disruption—often combined with persistent biofilm formation—drives non-resolving inflammation, impairs re-epithelialisation, and increases antimicrobial tolerance. As antibiotic resistance escalates, these features strengthen the rationale for microbiome-directed strategies that target wound ecology while reducing reliance on conventional antimicrobials. Current evidence is still dominated by mechanistic and preclinical studies, with only early clinical signals for selected approaches; therefore, next-generation probiotics, including *Lactiplantibacillus*/*Lactobacillus* spp., as well as defined prebiotic and postbiotic formulations, should be interpreted as promising adjuncts rather than clinically established therapies. Causal mechanisms, optimal formulations, reproducibility, and patient-level determinants of response remain insufficiently defined, representing a critical knowledge gap that limits translation. Here, we synthesise current evidence linking microbial ecology to key wound-healing pathways and propose a precision framework that integrates metagenomics, transcriptomics, metabolomics, and spatial profiling to map host–microbe interactions, identify predictive biomarkers, and guide stratified therapy. We further highlight combinatorial approaches pairing ecological engineering with biofilm-disruptive materials and immune-modulatory molecules. Realising the potential of these interventions will require mechanism-resolved clinical trials, standardised outcome frameworks, and patient stratification tools—advances that could improve chronic wound management while reducing selective pressure for antimicrobial resistance.

## 1. Introduction

Chronic wounds—including diabetic foot ulcers, venous leg ulcers, and pressure injuries—represent a major and growing clinical challenge, with profound impacts on quality of life and healthcare costs [1]. Despite advances in debridement, dressings, and antimicrobial protocols, durable healing remains difficult to achieve in a substantial fraction of patients, particularly when systemic comorbidities such as diabetes, peripheral vascular disease, or immunosuppression converge with local infection and impaired perfusion [1,2]. Understanding how the skin microbiome shapes barrier function and immune responses is therefore essential for interpreting its role—and therapeutic potential—in wound healing.

The skin is a highly specialised barrier continuously shaped by resident microorganisms. Culture-independent profiling has established that the cutaneous microbiome is a niche-structured ecosystem dominated by Actinobacteria, Firmicutes, Proteobacteria, and Bacteroidetes, with strong topographical variation and substantial inter-individual heterogeneity [3]. Beyond passive colonisation, skin commensals actively reinforce barrier function and regulate immune tone through antimicrobial activity and immunoregulatory signalling, influencing both innate sensing and tissue-adaptive programs [4].

A key hallmark of non-healing wounds is community disruption at the wound surface, frequently coupled with biofilm formation. Biofilms create a protected, polymicrobial state characterised by increased antimicrobial tolerance, immune evasion, and sustained inflammatory stimulation, collectively delaying re-epithelialisation and granulation tissue maturation [5]. Large-scale sequencing studies further support that chronic wounds harbour complex microbial communities whose composition and functional potential associate with wound type and clinical trajectories, underscoring that infection in chronic wounds is often a community-level phenomenon rather than a single-pathogen event [5,6]. The global rise in antimicrobial resistance further constrains conventional management, strengthening the rationale for antibiotic-sparing strategies that modify wound ecology while supporting host-directed resolution pathways.

Mechanistic studies have begun to define how commensals can promote repair, including keratinocyte-intrinsic signalling pathways that dampen excessive inflammation and adaptive immune programs that couple antimicrobial defence with tissue repair [7,8]. These insights motivate microbiome-targeted interventions—probiotics, prebiotics, and postbiotics—as adjuncts to standard care. However, the translational status of these interventions differs substantially: many mechanisms are supported primarily by in vitro and animal data, whereas clinically validated outcomes remain limited to a small number of early studies in selected wound types. Causal mechanisms, optimal formulations, reproducibility, and patient-level determinants of response remain insufficiently resolved, representing a knowledge gap that must be addressed to advance the field. Here, we synthesize current evidence linking microbial ecology to core wound-healing pathways and outline a precision framework integrating metagenomics, transcriptomics, metabolomics, and spatial profiling to map host–microbe crosstalk, identify predictive biomarkers, and guide stratified combination therapy that pairs ecological engineering with biofilm-disruptive materials and immune-modulatory molecules. This review is primarily intended for clinical researchers, microbiome scientists, and translational wound-healing researchers seeking to integrate microbial ecology, host immune mechanisms, and therapeutic development.

## 2. Skin Microbiome Composition

Culture-independent studies, accelerated by the Human Microbiome Project and subsequent large-scale surveys, established that the skin microbiome is a highly niche-structured ecosystem with marked interpersonal heterogeneity [9,10]. Community assembly is largely dictated by local microenvironmental constraints—sebum content, hydration, pH, temperature, and exposure—resulting in reproducible topographical signatures across sebaceous, moist, and dry sites [3,9]. At the phylum level, bacterial communities are dominated by four major phyla, each with distinct functional roles at the skin surface [3]. Actinobacteria, principally represented by *Cutibacterium* spp. in sebaceous regions, produce short-chain fatty acids and antimicrobial compounds that lower surface pH and inhibit pathogen colonisation—functions that are disrupted when wound exudate shifts local chemistry. Firmicutes, dominated by *Staphylococcus* and *Bacillus* spp., are among the most wound-relevant taxa: commensal staphylococci (e.g., *S. epidermidis*) actively suppress pathogenic competitors and modulate keratinocyte immune responses, while pathogenic strains (e.g., *S. aureus*) are the most frequently isolated organisms in non-healing wounds [3,5]. Proteobacteria encompass opportunistic genera—including *Pseudomonas*, *Klebsiella*, and *Acinetobacter*—that are typically low-abundance commensals on healthy skin but can rapidly expand to dominance in chronic, inflamed, or antibiotic-exposed wound environments [6]. Bacteroidetes contribute anaerobic fermentation capacity and protease activity, and their enrichment in deeper wound niches has been associated with proteolytic degradation of the extracellular matrix and impaired healing trajectories [11]. Understanding the baseline functional roles of these phyla is essential for interpreting their behaviour after injury: the wound surface is not colonised de novo but is restructured from a pre-existing community whose protective functions are progressively lost as dysbiosis deepens.

Beyond spatial organisation, the skin microbiome also exhibits distinct temporal dynamics shaped by environmental exposure and host physiology. Relatively stable niches (e.g., inguinal folds) often show lower within-site diversity, whereas highly exposed sites (e.g., palms) exhibit greater volatility and higher diversity, consistent with stronger environmental filtering and frequent microbial turnover [9]. Importantly, intra-individual variability over time should not be overlooked: local microenvironments within the same individual can diverge substantially depending on mechanical stress, moisture accumulation, and inflammatory status—factors directly relevant to wound-prone anatomical sites such as the plantar foot or sacrum. Host-intrinsic factors further shape these patterns: early-life microbial acquisition is moulded by delivery mode and environmental exposure, host genetics related to barrier function can associate with differences in microbial composition, and age- and sex-associated physiological changes—including pubertal shifts in sebum—contribute to variability across the lifespan [12,13].

Beyond bacteria, the healthy cutaneous ecosystem includes fungi, viruses, and mites, each with site-specific distributions and distinct functional relationships with the bacterial community. *Malassezia* spp. dominate the fungal microbiome of sebaceous sites and interact with keratinocytes and bacteria to modulate inflammatory and antimicrobial gene programs [14,15]. Demodex mites colonise sebaceous follicles and may influence local immune tone, particularly in the context of inflammatory skin disease. Bacteriophages—the viral component of the skin microbiome—can shape bacterial community structure through predation and horizontal gene transfer, with potential consequences for the spread of antimicrobial resistance genes across wound communities. Collectively, these ecological principles explain why even localised disruptions in barrier structure can precipitate rapid and sometimes irreversible community restructuring: disruption of barrier architecture, exudate-rich conditions, altered oxygenation, and frequent antimicrobial exposure can favour pathogenic consortia and biofilm formation—features repeatedly associated with impaired healing trajectories in chronic wounds [4].

Key message: The skin microbiome is not a static background feature but a dynamic, functionally active ecosystem whose disruption upon wounding sets in motion the ecological cascades that underpin chronic non-healing—making it a rational and tractable therapeutic target.

## 3. Skin Microbiome-Mediated Immune Modulation and Protection Against Pathogens

At the host interface, microbial ligands are sensed by keratinocytes and immune cells through pattern-recognition receptors (PRRs), integrating microbial cues into wound-relevant inflammatory programs. A paradigmatic example is staphylococcal lipoteichoic acid (LTA), which signals via TLR2 on keratinocytes to suppress TLR3-driven inflammation after injury, thereby limiting excessive cytokine release while maintaining effective repair [7].

The compositional and ecological features described above are not merely structural: they translate directly into functional immune consequences at the skin barrier, particularly in the context of injury and wound repair. The cutaneous microbiome is an active determinant of barrier immunity, shaping both early-life immune calibration and adult tissue homeostasis. Commensal staphylococci contribute to colonisation resistance by producing antimicrobial molecules, including phenol-soluble modulins (PSMs), which selectively inhibit pathogenic competitors while preserving resident community structure [16]. At the host interface, microbial ligands are sensed by keratinocytes and immune cells through pattern-recognition receptors (PRRs), integrating microbial cues into wound-relevant inflammatory programs. A paradigmatic example is staphylococcal lipoteichoic acid (LTA), which signals via TLR2 on keratinocytes to suppress TLR3-driven inflammation after injury, thereby limiting excessive cytokine release while maintaining effective repair [7].

Beyond modulating innate sensing, commensals also shape adaptive immune programs in a context-dependent and largely non-inflammatory manner—a capacity with direct relevance to the wound-healing trajectory. Colonisation with *Staphylococcus epidermidis* induces tissue-resident IL-17A^+^ CD8^+^ T cells that localise to the epidermis and enhance barrier protection against pathogen invasion, illustrating how commensals can amplify protective immunity without overt pathology [17]. In parallel, commensal and pathogenic staphylococci differ in their capacity to activate keratinocyte signalling and induce antimicrobial peptide (AMP) expression, highlighting that “microbial identity” and “signal quality” are critical variables in cutaneous immune outcomes [18]. Host-derived AMPs (e.g., β-defensins and cathelicidin LL-37) constitute a major effector layer of innate defence; their expression can be induced in keratinocytes upon bacterial exposure and contributes to activity against *Staphylococcus aureus*, including resistant strains [18,19].

Beyond direct antimicrobial effects, specific commensal strains can protect against inflammatory disease states and malignant transformation. In atopic dermatitis, human commensals produce antimicrobial factors that suppress *S. aureus* colonisation and are deficient in affected skin, supporting a functional loss of colonisation resistance in disease [20]. Moreover, some *S. epidermidis* strains produce 6-N-hydroxyaminopurine (6-HAP), a metabolite that inhibits DNA polymerase activity in tumour cell lines and reduces tumour growth in experimental models, suggesting a broader role for commensal metabolites in cutaneous immune surveillance [19]. Extending beyond bacteria, *Malassezia* spp. can modulate keratinocyte inflammatory and antimicrobial gene programs, reinforcing the concept that fungi may also participate in shaping cutaneous immune tone in a niche-dependent manner [14,15].

Collectively, these observations support a mechanistic framework in which commensals promote barrier integrity and immune resolution through layered antimicrobial activity, PRR-dependent tuning of inflammation, and induction of protective tissue-resident lymphocyte programs. Dysbiosis and biofilm formation in chronic wounds can therefore be viewed as a failure of these regulatory circuits, shifting the system toward persistent inflammation, impaired clearance, and delayed re-epithelialisation [4]. To improve readability and mechanistic integration, Table 1 summarises key commensal microorganisms, their associated signalling pathways, target cell types, functional outcomes, and relevance to wound healing.

Key message: Commensals do not merely coexist with the host immune system—they actively calibrate it. Understanding the specific molecular interactions through which skin microorganisms promote repair or sustain pathology is the essential prerequisite for designing targeted, mechanism-based interventions.

### 3.1. The Complex Interplay of Skin Microbiota and Host Immune Response in Wound Recovery

Cutaneous wound repair is a clinically relevant and mechanistically tractable system in which host–microbe interactions can be interrogated across distinct healing trajectories. While acute wounds typically progress through a coordinated sequence of haemostasis, inflammation, proliferation and remodelling, chronic wounds are defined by failure to transition from inflammation to resolution, with consequent defects in re-epithelialisation and matrix restoration [2,22]. Microbial ecology is not a passive bystander in this process: injury reshapes the wound surface into a nutrient-rich, heterogeneous microenvironment whose altered physical and chemical conditions profoundly influence both immune responses and microbial behaviour [6,23].

Oxygen gradients are among the most biologically consequential features of the wound microenvironment. The wound bed is typically hypoxic at its centre, with oxygen tension falling below 10 mmHg in necrotic or heavily exudating tissue, compared to approximately 40–60 mmHg in healthy dermis. This hypoxia impairs neutrophil oxidative burst and reduces macrophage bactericidal capacity, directly weakening the host’s ability to clear pathogens. At the same time, low oxygen favours the growth of anaerobic taxa—including *Bacteroides*, *Fusobacterium*, and *Peptostreptococcus* spp.—that are poorly targeted by aerobically oriented antimicrobials and contribute to protease-rich, matrix-degrading environments [2,6,24].

pH shifts further restructure both microbial communities and immune cell function. Healthy skin maintains a surface pH of approximately 4.5–5.5, which suppresses pathogen growth and supports commensal-dominated communities. In chronic wounds, exudate accumulation, bacterial metabolic activity, and tissue necrosis elevate pH to 7.0–8.9, a range that favours *P. aeruginosa* and *Proteus* spp. growth, increases the activity of matrix metalloproteinases (MMPs) that degrade growth factors and extracellular matrix proteins, and impairs the function of endogenous AMPs such as LL-37 whose antimicrobial activity is pH-dependent [2,6,24].

Moisture dysregulation—either excessive exudation or desiccation—disrupts the physical barrier and alters the nutrient landscape of the wound surface. High exudate levels create conditions that dilute growth factors, increase protease burden, and provide a nutrient-rich medium for bacterial proliferation and biofilm maturation [6,23]. In contrast, dry wound environments impair keratinocyte migration and re-epithelialisation.

Immune pressure—mediated by sustained neutrophil infiltration, reactive oxygen species, proteases, and pro-inflammatory cytokines—selectively favours microorganisms with resistance mechanisms (e.g., catalase-positive *S. aureus*, protease-secreting *P. aeruginosa*) while damaging commensal taxa less adapted to oxidative or proteolytic stress. These conditions drive rapid community restructuring and, in susceptible hosts, can stabilise dysbiotic states that perpetuate inflammatory pathology [6,23]. Taken together, the wound microenvironment acts as a powerful ecological filter—not simply a consequence of infection, but an active determinant of which microbial communities persist and how host immunity responds.

### 3.2. Ecological Shifts After Injury: From Commensal Buffering to Dysbiosis

In acute repair, early microbial signals can support “colonisation resistance” and limit pathogen expansion through direct antimicrobial effects and by calibrating epithelial and immune responses [3,4]. In contrast, chronic wounds often exhibit persistent ecological disturbance driven by repeated debridement, topical antiseptics/antibiotics, ischemia, diabetes-associated immune dysfunction, and sustained exudation, factors that collectively shift the wound ecosystem toward opportunistic consortia and biofilm-prone communities [2,6,24]. Importantly, these states are frequently polymicrobial, and the clinical phenotype may reflect community-level behaviour rather than the presence of a single pathogen [5,11].

### 3.3. Innate Sensing Sets the Inflammatory “Set-Point” of Repair

Keratinocytes, neutrophils, macrophages and other resident cells integrate microbial cues via PRRs, shaping downstream cytokine circuits that determine whether inflammation resolves or persists. A canonical example is the capacity of commensal signals to tune keratinocyte PRR responses: commensal-derived ligands can dampen excessive inflammation following injury by modulating TLR-dependent pathways, thereby limiting tissue-damaging inflammation while preserving barrier defence [7]. In parallel, commensals can instruct protective adaptive programs. Colonisation with *Staphylococcus epidermidis* promotes skin-resident IL-17A^+^ CD8^+^ T cells that enhance barrier immunity and antimicrobial protection [17]. In the context of injury, these commensal-instructed pathways can support a timely shift from pathogen control toward epithelial proliferation and re-epithelialisation [8].

Microbial metabolites also contribute to immune tuning at the wound interface. Short-chain fatty acids and related products can act on keratinocytes and immune cells to modulate inflammatory outputs, although their net effect is context-dependent and likely shaped by local concentrations, receptor expression, and concurrent tissue damage signals [21]. Together, these data support a model in which the microbiome influences the “inflammatory set-point” of repair by affecting keratinocyte signalling, leukocyte recruitment, and the balance between antimicrobial defence and pro-resolving programs [3,4].

### 3.4. Chronic Wounds: Failure of Resolution and Immune Deviation

A defining feature of chronic wounds is non-resolving inflammation with sustained neutrophil infiltration, protease burden, oxidative stress, and impaired macrophage reprogramming toward pro-repair phenotypes [2,22]. This failure of resolution compromises granulation tissue formation, angiogenesis and re-epithelialisation, and increases susceptibility to persistent microbial communities [2]. At the same time, chronic wounds often display prolonged exposure to inflammatory cytokines and damage-associated signals that further remodel the microbial niche, favouring organisms adapted to inflammatory and protease-rich environments [6,24].

### 3.5. Biofilms as Ecological Lock-In States That Sustain Chronicity

Biofilm formation represents a key mechanism linking microbial ecology to chronic inflammation. Biofilms are structured communities embedded in an extracellular polymeric matrix that increases antimicrobial tolerance and protects microbes from effective clearance [23,25]. Clinically, biofilms are markedly more prevalent in chronic wounds than in acute wounds, and their presence correlates with stalled healing trajectories [23]. Mechanistically, biofilms can sustain inflammatory activation through persistent antigenic stimulation, complement activation, and “frustrated phagocytosis”, while simultaneously limiting antibiotic penetration and enabling cooperative polymicrobial behaviour [25,26]. Recent reviews further reinforce this concept by showing that biofilms interact directly with innate immune pathways, sustaining non-resolving inflammation, immune evasion, and biofilm-associated antimicrobial tolerance in chronic wounds [27,28]. High-throughput sequencing studies of chronic wounds further highlight frequent dominance of *Staphylococcus* spp. and opportunistic Gram-negative taxa (notably *Pseudomonas*), often with anaerobes enriched within protected micro-niches, consistent with strong spatial heterogeneity in oxygen and nutrient availability across the wound bed [5,11].

### 3.6. Clinical Signatures, Prediction, and Context Dependence

Clinical microbiome studies indicate that wound type, host factors and anatomical site shape community structure and functional potential, and that microbial profiles can associate with healing outcomes [3,5,29]. Recent longitudinal profiling of diabetic ulcers further supports this association, showing that the healing process correlates with measurable shifts in cutaneous microbiota composition [30]. For example, *S. aureus* is repeatedly identified among prevalent wound-associated taxa, including diabetic foot ulcers where methicillin-resistant strains may occur and complicate management [31,32]. Longitudinal and site-matched studies also suggest that baseline skin microbiota may influence susceptibility to later ulceration in high-risk feet, supporting the concept that microbial ecology can contribute to risk stratification in vulnerable populations [33].

Notably, host–microbe interactions in wound repair are context-dependent and may appear paradoxical across experimental systems. Germ-free or microbiota-reduced settings can accelerate certain aspects of closure in murine models, likely reflecting reduced neutrophil-driven tissue damage under specific conditions; however, these results must be interpreted alongside the protective roles of commensals in pathogen resistance and immune calibration [34,35]. The key translational implication is that beneficial versus detrimental microbial effects are shaped by the balance between antimicrobial defence and collateral inflammatory injury, the spatial organisation of microbes (planktonic vs. biofilm), and the host’s capacity to execute resolution programs [2,6].

Collectively, these findings support reframing chronic wounds as disorders of coupled ecology and immunity. Microbial dysbiosis and biofilm formation are not merely markers of infection but active drivers of inflammatory persistence and impaired epithelial repair. This framework strengthens the rationale for combination strategies that (i) disrupt biofilms and community structure, (ii) recalibrate epithelial and immune signalling toward resolution, and (iii) engineer the wound ecosystem to restore protective functions while minimising antibiotic selection pressure [2,3,6,25].

Key message: Chronic wounds should be understood as disorders of coupled ecology and immunity, not simply as infected tissue. This reframing shifts the therapeutic target from microbial eradication to ecological restoration and immune recalibration—goals for which prebiotics, probiotics, and postbiotics are specifically designed.

### 3.7. Prebiotics, Probiotics, and Postbiotics: Innovative Approaches for Managing Chronic Wound Infections

The therapeutic landscape for chronic wounds has historically been dominated by debridement, offloading, compression, and antimicrobial dressings. While these approaches address local tissue quality and bacterial burden, they do not resolve the underlying ecological dysfunction that sustains non-healing phenotypes, namely, the loss of colonisation resistance, the persistence of pathogen-dominant biofilm communities, and the failure of host immune circuits to transition from inflammation to repair. Standard-of-care antimicrobials, including topical antiseptics (e.g., silver, iodine, chlorhexidine) and systemic antibiotics, can reduce bioburden acutely but often fail to eradicate biofilms, select for resistant strains, and do not restore a balanced microbial community [26,36]. This gap has driven growing interest in microbiome-targeted therapeutics—prebiotics, probiotics, and postbiotics—as mechanistically distinct, antibiotic-sparing adjuncts designed to simultaneously address ecology, biofilm architecture, and host immune reprogramming.

To contextualize these approaches, it is important to understand what conventional treatment cannot achieve. Topical antiseptics such as silver-containing dressings reduce surface bioburden but have limited penetration into established biofilm matrices and can impair keratinocyte viability at higher concentrations [26,36]. Systemic antibiotics are effective against planktonic bacteria but are 100–1000-fold less effective against biofilm-embedded organisms, and broad-spectrum use accelerates AMR gene selection—a problem of growing clinical urgency, particularly in diabetic foot infections where multidrug-resistant organisms are increasingly prevalent [31,32]. Recent systematic evidence in infected diabetic foot ulcers confirms the clinical relevance of antimicrobial resistance patterns, reinforcing the need for adjunctive antibiotic-sparing strategies that do not rely exclusively on conventional antimicrobial pressure [37]. Against this background, microbiome-directed interventions offer a complementary mechanism of action: rather than killing bacteria non-selectively, they aim to restructure the ecological balance of the wound community, fortify host innate defences, and accelerate the transition to a pro-repair inflammatory state.

**Figure 1:**
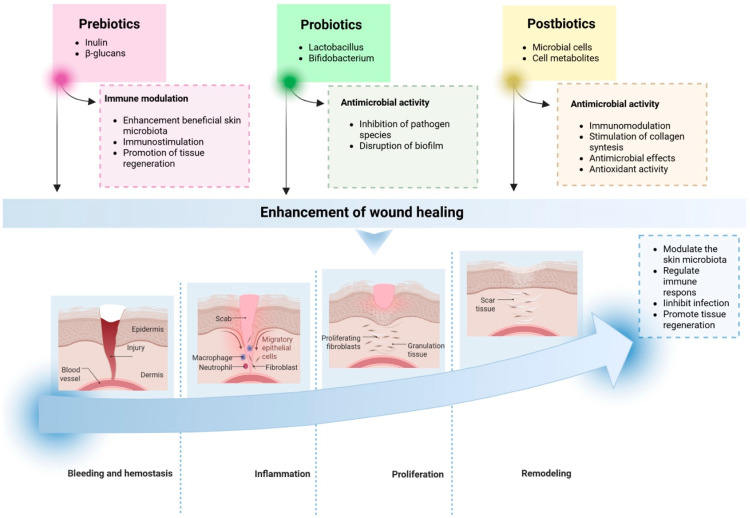
Prebiotics, probiotics, and postbiotics as complementary microbiome-targeted tools to support cutaneous wound healing. The upper panel illustrates the principal mechanisms of action of each intervention class at the wound surface. Prebiotics, such as β-glucans and oligosaccharides, may enrich protective commensals and engage innate immune pathways; probiotics, such as Lactiplantibacillus plantarum, may competitively exclude pathogens, produce antimicrobial metabolites, interfere with biofilm formation, and modulate macrophage and keratinocyte signalling; postbiotics, including inanimate microbial preparations, bacteriocins, and conditioned secretions, may deliver defined immunomodulatory and antimicrobial effectors without the viability constraints of live preparations. The lower panel maps these mechanisms onto the main phases of wound repair—haemostasis, inflammation, proliferation, and remodelling—highlighting their potential contribution to immune resolution, microbial control, tissue regeneration, and durable closure. All three modalities should be interpreted as adjuncts to standard wound care rather than stand-alone therapies.

Microbiome-targeted therapeutics, including prebiotics, probiotics, and postbiotics, are being explored as mechanistically distinct routes to reshape wound communities, attenuate maladaptive inflammation, and improve repair outcomes [38]. A recent scoping review of probiotic, prebiotic, synbiotic, and postbiotic cutaneous formulations also highlights the growing interest in these approaches for wound care, while emphasizing the heterogeneity of formulations, outcomes, and evidence quality across studies [39]. At present, their evidentiary maturity is uneven: prebiotic and postbiotic concepts are supported largely by mechanistic and preclinical datasets, whereas probiotic/live-biotherapeutic approaches include a limited number of clinical studies but still lack broad, reproducible efficacy validation. Importantly, these interventions should be framed as adjuncts to standard wound care, for example, debridement, off-loading and perfusion optimisation, with the explicit goal of (i) reducing pathogenic dominance and biofilm stability, (ii) restoring colonisation resistance, and (iii) reprogramming inflammatory circuits toward pro-repair trajectories. Accordingly, Figure 1 summarises how prebiotics, probiotics, and postbiotics may act as complementary microbiome-targeted tools at the wound surface, linking their ecological, antimicrobial, and immunomodulatory mechanisms to specific phases of cutaneous wound repair.

### 3.8. Prebiotics: Ecological Substrates to Promote Protective Skin Commensals

Prebiotics are traditionally defined as substrates selectively utilised by host microorganisms that confer a health benefit [38]. In a wound or peri-wound context, topical prebiotic concepts aim to favour protective commensals, for example, coagulase-negative staphylococci, and suppress opportunistic pathogens, for example, *S. aureus* and *Pseudomonas* spp., through nutrient competition and metabolite-driven growth inhibition. In vitro work supports that specific oligosaccharides and polyols can differentially modulate *S. epidermidis* versus *S. aureus*, with downstream effects on antibiofilm activity; nevertheless, direct clinical validation in chronic wounds remains sparse, and benefits cannot yet be assumed across wound types or patient populations.

Among immunologically active polysaccharides, β-glucans are frequently discussed as prebiotic-like bioactives in wound settings because they can engage innate immune receptors (notably Dectin-1) and modulate macrophage-driven repair programs [40]. In clinical translation, soluble β-1,3/1,6-glucan has been evaluated as a topical adjunct for diabetic foot ulcers in a randomised, double-blind, placebo-controlled phase II study. While mechanistic granularity in humans remains limited, the translational appeal lies in coupling immune modulation (resolution support) with ecosystem effects (microbial burden/biofilm stability), ideally measured using longitudinal microbiome and inflammatory biomarkers.

Prebiotic studies in wounds often lack strain-level readouts, metabolite profiling, and standardised endpoints, including biofilm burden, inflammatory mediators and time-to-closure. Future trials should integrate microbiome-aware outcomes and define whether benefits arise from ecological shifts, host immune reprogramming, or both. A further limitation is that broad 16S rRNA profiling can miss strain-level and functional changes relevant to nutrient utilisation, antimicrobial resistance, and metabolite production; therefore, shotgun metagenomics or targeted functional assays should be incorporated where feasible.

### 3.9. Postbiotics: Inanimate Microbial Preparations with Defined Bioactive Components

To strengthen conceptual clarity, the term ‘postbiotic’ should follow the ISAPP consensus definition: “a preparation of inanimate microorganisms and/or their components that confers a health benefit on the host.” Postbiotics encompass a broad set of effectors relevant to wounds, including cell wall components (e.g., peptidoglycan, teichoic acids), surface proteins, extracellular polysaccharides, and secreted antimicrobials (e.g., bacteriocins), provided they are delivered as inanimate preparations with demonstrable benefit [41]. Recent evidence syntheses further position postbiotics as microbial-derived therapeutic candidates for wound-healing disorders, but also underline that their clinical translation depends on clearer molecular definition, potency assays, dosing strategies, and mechanism-linked outcome measures [42].

Mechanistically, postbiotics can be positioned in two complementary therapeutic lanes (Figure 1), although most evidence in wound healing remains preclinical and requires validation with defined composition, dose, potency, and mechanism-linked biomarkers. First, immune calibration (resolution support): selected microbial-associated molecular patterns can engage PRRs and reshape cytokine output, potentially reducing excessive TNF/IL-1β/IL-6 signalling while supporting pro-repair mediators (e.g., IL-10/TGF-β), depending on context and dose. The key advantage over live biotherapeutics is improved stability and safety (manufacturing control, storage, and reduced risk in immunocompromised hosts) [41]. Second, direct antimicrobial/anti-biofilm activity: bacteriocins and related peptides can disrupt pathogen membranes, and extracellular polymers may interfere with adhesion and early biofilm architecture—properties that are attractive when combined with debridement and topical anti-biofilm materials [43].

Most postbiotic wound claims in the literature remain preclinical and heterogeneous, with variable preparations, undefined dose or potency, and limited mechanistic readouts. Stronger studies should specify the following: composition, using omics and biochemical characterisation; potency assays, including anti-biofilm activity and AMP induction; mechanism-linked biomarkers in vivo; and clinically relevant endpoints such as durable closure, recurrence, pain, odour, and infection control.

### 3.10. Probiotics and Live Biotherapeutics: Competitive Exclusion, Biofilm Interference, and Host–Pathway Modulation

Probiotics are classically defined as live microorganisms that, when administered in adequate amounts, confer a health benefit on the host [44]. In wound contexts, probiotics may act via the following: (i) competitive exclusion (niche occupation, nutrient competition), (ii) anti-pathogen effectors (organic acids, bacteriocins, AMP induction), (iii) biofilm interference, and (iv) host immune pathway modulation (PRR tuning, inflammasome regulation, macrophage programming) [6] (Figure 1).

Human evidence remains limited but is emerging most clearly for topical application in diabetic foot ulcers. A randomised controlled trial in outpatients with complicated diabetic foot ulcers evaluated surgical debridement alone versus debridement plus topical *Lactiplantibacillus plantarum* (ATCC 10241), supporting feasibility and a clinical signal as an adjunct approach rather than definitive evidence of efficacy across chronic wounds. Mechanistic work in parallel suggests that *L. plantarum* can influence inflammatory pathways relevant to chronicity, including inflammasome-linked signalling, and thereby impact repair trajectories, but these mechanisms still require confirmation in longitudinal, biomarker-informed human studies.

However, translating probiotics into robust wound therapeutics requires tighter control of formulation variables: strain identity, ideally genome-verified; viable dose at application; vehicle compatibility; stability over time; and interactions with antiseptics, antibiotics and dressings. Moreover, patient heterogeneity, including ischemia, glycaemic control, neuropathy and immunosuppression, as well as local biofilm architecture and prior antimicrobial exposure, likely determines response, arguing for a precision framework where baseline microbiome state and inflammatory tone guide selection of strain or cocktail, delivery route, and co-interventions [9,10].

Taken together, prebiotics, probiotics, and postbiotics represent mechanistically distinct but potentially complementary tools for chronic wound management. Prebiotics (e.g., inulin, β-glucans) may selectively enrich protective commensals and modulate innate immune receptor engagement; probiotics (e.g., *Lactiplantibacillus* spp.) can competitively exclude pathogens, disrupt early biofilm formation, and reprogram macrophage-centred repair programs; postbiotics deliver defined inanimate effectors—cell wall components, bacteriocins, secreted metabolites—with favourable stability and safety profiles. Nevertheless, it is critical to note that the evidence supporting these benefits is predominantly preclinical. The few available clinical studies are limited in sample size, wound-type diversity, and mechanistic resolution, and do not yet permit broad efficacy claims across chronic wound populations. Their role in clinical practice should therefore be interpreted as adjunctive and investigational pending larger, mechanism-resolved trials. A comparative overview of these three modalities is provided in Table 2.

### 3.11. Toward Precision Microbiome Therapy: What Wound Trials Should Measure

The translation of microbiome-targeted interventions into clinical practice requires more than demonstration of efficacy in single-endpoint trials. Chronic wounds are heterogeneous in their microbial composition, inflammatory state, host comorbidities, and treatment history—variables that interact to determine whether any given intervention will produce benefit, no effect, or harm in a specific patient. Current clinical trials in this space are limited by small sample sizes, lack of pre-specified microbiome endpoints, short follow-up periods, and absence of mechanistic stratification, making it difficult to identify which patients benefit, through which mechanisms, and under what wound conditions [5,6]. Advancing the field therefore requires a shift toward mechanism-resolved, biomarker-stratified trial designs.

To achieve this, wound studies should move beyond single clinical endpoints (e.g., faster closure) and adopt mechanism-resolved outcome sets. These should include: (i) longitudinal, strain-resolved microbiome profiling to capture community dynamics and pathogen/commensal turnover; (ii) spatial mapping of microbial organisation (planktonic vs. biofilm) alongside inflammatory cell infiltration within the wound bed; (iii) functional readouts of microbial and host activity, including metabolite signatures (e.g., organic acids) and enzymatic/proteolytic activity; and (iv) host biomarkers of inflammatory resolution and repair competence, such as macrophage phenotypic states and cytokine balance (e.g., IL-1β/TNF versus IL-10/TGF-β). Mechanism-informed combination regimens that integrate ecological engineering with biofilm-disruptive materials and immune-modulatory molecules are likely to yield the most robust benefit, particularly in advanced chronic wounds where biofilms stabilise dysbiotic communities and sustain non-resolving inflammation [45].

To illustrate how a multi-omics precision framework could be operationalised, consider a patient with a non-healing diabetic foot ulcer of more than four weeks’ duration. At baseline, wound swab or biopsy material would undergo 16S rRNA sequencing (or, where capacity exists, shotgun metagenomics) to define community composition at genus and, ideally, strain level, flagging dominance by *S. aureus*, *P. aeruginosa*, or anaerobic consortia. Wound exudate would be profiled for inflammatory mediators (IL-1β, TNF-α, IL-10, MMP-8/9) and, where available, metabolomics analysis would identify short-chain fatty acid signatures reflecting fermentative activity of the microbial community. Recent clinical profiling studies integrating skin microbiome and metabolome data during re-epithelialisation support this approach, suggesting that combined microbial and metabolic readouts may help link community dynamics to repair trajectories and treatment response [REF]. Tissue biopsy or biofilm swabs could be used for fluorescence in situ hybridisation (FISH) or confocal imaging to spatially map biofilm architecture relative to the wound margin. Integration of these datasets would stratify patients into ecological subtypes—for example, biofilm-dominant with Gram-positive pathogen overgrowth versus polymicrobial anaerobic community—and guide selection of the microbiome-targeted adjunct most likely to be beneficial. Serial sampling at weeks two, four, and eight would then enable resolution tracking and allow early identification of non-responders.

It is important to distinguish what is currently actionable from what remains a future goal. The following are currently feasible in well-resourced clinical trial settings: 16S amplicon sequencing of wound swabs, ELISA-based exudate cytokine profiling (IL-1β, IL-10, MMP-8), and semi-quantitative biofilm detection by confocal microscopy or plate-based assays. The following are under active development but not yet standardised: shotgun metagenomics integrated with metabolomics in wound-specific pipelines, spatial transcriptomics of wound-margin tissue, and metatranscriptomics for active gene expression profiling of the wound microbiome. The following are aspirational: real-time, point-of-care microbiome diagnostics to guide bedside therapeutic decisions; fully integrated multi-omics platforms that couple ecological readouts with host immune phenotyping for automated patient stratification.

Key message: Mechanistic, multi-omics trial designs are not optional enhancements—they are necessary conditions for identifying which patients benefit from microbiome-targeted interventions and why. Without them, the field risks accumulating inconclusive results that neither confirm nor refute the therapeutic potential of these approaches.

### 3.12. Methodological Limitations and Challenges in Reproducibility

Despite substantial progress, the evidence base for microbiome-targeted interventions in chronic wound healing is constrained by several interrelated methodological limitations that must be explicitly acknowledged when interpreting the existing literature.

**Variability in microbiome profiles across studies.** Reported microbiome composition in chronic wounds shows considerable inconsistency across studies, reflecting genuine biological heterogeneity (wound type, anatomical site, comorbidities, prior antimicrobial exposure) as well as technical variation in sampling protocols, DNA extraction methods, sequencing platforms, and bioinformatic pipelines. While *Staphylococcus* spp. and *Pseudomonas* spp. are frequently identified as dominant taxa in chronic wounds [5,11], their relative abundance and co-occurrence patterns differ substantially between diabetic foot ulcers, venous leg ulcers, and pressure injuries. This heterogeneity complicates cross-study comparisons and limits the generalisability of conclusions drawn from any single cohort.

**Limitations of 16S rRNA sequencing.** The majority of wound microbiome studies rely on 16S rRNA gene amplicon sequencing, which provides genus- or species-level taxonomic assignments but cannot resolve strain-level variation, horizontal gene transfer events, antimicrobial resistance gene carriage, or the functional metabolic potential of the community [5,11]. These limitations are clinically relevant: two strains of the same species may differ profoundly in virulence, biofilm-forming capacity, and susceptibility to treatment. Shotgun metagenomics enables functional annotation and strain-level resolution but requires greater sequencing depth and computational resources, and introduces its own challenges in reference database coverage for skin and wound taxa. A recent review of direct metagenomics in non-surgical hard-to-heal wounds reinforces this limitation, highlighting the value of metagenomic approaches for broader pathogen detection, functional inference, and antimicrobial-resistance surveillance, while also noting the need for standardised workflows before routine clinical implementation [46]. The field would benefit from a phased approach in which 16S profiling is used for broad community characterisation and hypothesis generation, while shotgun metagenomics and metatranscriptomics are incorporated in mechanism-focused studies and clinical trials.

**Reproducibility and standardisation across trials.** Published intervention trials—particularly those evaluating probiotics, prebiotics, and postbiotics—vary substantially in strain identity (often not genome-verified), preparation method, dose, vehicle, frequency and duration of application, concurrent wound care, and outcome measurement. Most studies lack pre-specified microbiome endpoints, do not report longitudinal changes in community composition or functional potential, and use short-term area reduction as the primary efficacy measure, without capturing biofilm burden, inflammatory mediator profiles, recurrence, or patient-reported outcomes. This heterogeneity renders meta-analytic synthesis unreliable and obscures whether reported benefits arise from ecological restructuring, host immune reprogramming, or non-specific wound care effects. Consensus on minimum reporting standards for microbiome-informed wound trials—including standardised sampling, sequencing, and bioinformatic workflows, as well as mechanism-linked secondary endpoints—is urgently needed to enable reproducible, cross-comparable evidence generation.

## 4. Future Perspectives

Over the last decade, microbiome-informed approaches for chronic wounds have progressed from descriptive dysbiosis surveys to mechanistically motivated interventions designed to reshape wound ecology and recalibrate host inflammatory–resolution programs. Chronic wounds frequently exhibit polymicrobial biofilms and community states that increase antimicrobial tolerance and sustain non-resolving inflammation, providing a clear rationale for adjunct, antibiotic-sparing strategies that target both microbial organisation and host–pathway dysfunction.

Current clinical evidence remains early-stage but supports feasibility and safety signals for selected microbiome-oriented interventions. Topical soluble yeast β-1,3/1,6-glucan has shown clinical benefit in diabetic foot ulcers in a randomised, double-blind, placebo-controlled phase II study, consistent with the hypothesis that immune training and macrophage-centred repair programs can be therapeutically leveraged. More broadly, β-glucans are mechanistically linked to Dectin-1 engagement and downstream macrophage activation, supporting angiogenesis and tissue repair in wound models. In parallel, live biotherapeutics (probiotics) and inanimate microbial preparations (postbiotics) are being investigated as complementary tools to (i) restore colonisation resistance, (ii) attenuate excessive PRR-driven cytokine signalling, and (iii) disrupt early biofilm formation or stability through secreted antimicrobials such as bacteriocins.

Despite these advances, the evidence base remains fragmented. Trials are heterogeneous in strain identity (often not genome-verified), dosing, formulation/vehicle, route of delivery, and concomitant wound care, limiting cross-study comparisons and meta-analytic synthesis. Outcome selection is frequently restricted to short-term area reduction, with limited assessment of durability (recurrence), scar quality, symptom burden (pain, odour), and patient-reported outcomes. Mechanistic resolution is additionally constrained by scarce longitudinal microbiome profiling (strain-level, functional potential) and limited integration of host inflammatory biomarkers, preventing discrimination between benefits driven by ecological restructuring, host immune reprogramming, or both.

Future studies should prioritise mechanism-informed, standardised trial frameworks. First, interventions should be designed as combination regimens—ecological engineering plus anti-biofilm materials and targeted immune modulation, reflecting the lock-in nature of biofilm-associated dysbiosis. Second, delivery systems (e.g., hydrogels, responsive dressings, electrospun scaffolds) should be optimised to ensure local persistence, controlled release, and compatibility with antiseptics and standard care. Third, multi-layer profiling (shotgun metagenomics, metatranscriptomics, metabolomics, and spatial approaches) should be embedded into trial design to map host–microbe crosstalk and enable biomarker-guided stratification. Finally, regulatory translation will require rigorous safety assessment, including monitoring of AMR gene dynamics and horizontal gene transfer potential, particularly for repeated or long-term use of live preparations. Recent studies and reviews published between 2020 and 2026 reinforce this direction across complementary domains, including biofilm–immune interactions and biofilm-associated tolerance [28], antimicrobial resistance in diabetic foot ulcers [37], wound microbiome dynamics during healing [30], the methodological transition from 16S rRNA profiling to shotgun metagenomics [46], microbiome–metabolome integration and multi-omics profiling [42], and emerging prebiotic, probiotic, and postbiotic wound-care strategies [39].Taken together, the next phase of the field should shift from proof-of-concept monotherapies toward stratified, mechanism-resolved combination strategies that integrate microbial ecology, biofilm biology, and host immune repair circuits to improve healing outcomes while reducing selective pressure for antimicrobial resistance.

## Figures and Tables

**Table 1:** Key commensal microorganisms and their host immune interactions relevant to skin barrier integrity and wound healing.

Microbial Species/Group	Signalling Pathway/Mechanism	Target Cell Type	Functional Outcome	Wound Relevance
*S. epidermidis* (PSMs)	Direct competitive exclusion; membrane disruption of pathogens	Commensal bacteria (community-level)	Selective inhibition of *S. aureus*; colonisation resistance	Loss of *S. epidermidis* dominance in chronic wounds allows pathogen overgrowth [16]
*S. epidermidis* (LTA via TLR2)	TLR2 signalling suppression of TLR3-driven inflammation	Keratinocytes	Dampened post-injury cytokine release; maintenance of barrier repair	Reduced commensal TLR2 signalling in dysbiotic wounds may contribute to inflammatory persistence [7]
*S. epidermidis* (colonisation)	Induction of tissue-resident lymphocytes	IL-17A+ CD8+ T cells (skin-resident)	Enhanced barrier protection; antimicrobial surveillance without overt inflammation	Absence of commensal-instructed tissue-resident immunity may impair pathogen control at wound margins [17]
*S. epidermidis* (6-HAP metabolite)	Inhibition of DNA polymerase	Tumour cells/epithelial cells	Suppression of abnormal proliferation; immune surveillance	Broader role of commensal metabolites in maintaining epithelial homeostasis around wounds [20]
Commensal staphylococci (general)	AMP induction (beta-defensins, LL-37)	Keratinocytes	Innate antimicrobial defence against S. aureus including MRSA	AMP deficiency in dysbiotic wounds reduces pathogen clearance capacity [18,20]
*Malassezia* spp.	Modulation of keratinocyte inflammatory and antimicrobial gene programs	Keratinocytes	Context-dependent pro- or anti-inflammatory immune tone	Fungal community disruption in wound ecology may alter keratinocyte responses [14,15]
Short-chain fatty acids (SCFA)	HDAC inhibition; FFAR2/3 receptor signalling	Keratinocytes; immune cells	Modulation of TLR-driven inflammatory outputs; tuning of innate immune set-point	SCFA gradients in wound exudate may influence whether inflammation resolves or persists [21]

**Table 2:** Comparison of prebiotic, probiotic, and postbiotic interventions for chronic wound management: mechanisms of action, level of evidence, and key advantages and limitations.

Feature	Prebiotics	Probiotics (Live Biotherapeutics)	Postbiotics
Definition	Selectively fermented substrates that confer benefit by modulating host microorganisms [38].	Live microorganisms that confer a health benefit when administered in adequate amounts [44].	Preparations of inanimate microorganisms and/or their components that confer a health benefit [41].
Examples in wound context	β-glucans, e.g., β-1,3/1,6-glucan; inulin; specific oligosaccharides and polyols.	*Lactiplantibacillus plantarum*; *Lactobacillus* spp.; topical application.	Bacteriocins; peptidoglycan fragments; teichoic acids; heat-inactivated cell preparations; conditioned supernatants.
Primary mechanisms of action	Selective enrichment of protective commensals; Dectin-1 engagement and macrophage activation; nutrient competition favouring colonisation resistance.	Competitive exclusion; bacteriocin and organic acid production; antimicrobial peptide induction; biofilm interference; pattern-recognition receptor tuning; inflammasome and macrophage modulation.	Pattern-recognition receptor-dependent immune calibration, including TNF/IL-1β suppression and IL-10/TGF-β support; direct antimicrobial and anti-biofilm activity; stability advantages over live preparations.
Highest level of evidence	Phase II randomized controlled trial with β-1,3/1,6-glucan in diabetic foot ulcers.	Single randomized controlled trial with *L. plantarum* in diabetic foot ulcers, supported by additional mechanistic and animal data.	Predominantly in vitro and animal studies; no published wound-specific randomized controlled trials to date.
Evidence quality	In vitro ✓/In vivo (animal) ✓/Clinical (early) ✓	In vitro ✓/In vivo (animal) ✓/Clinical (limited) ✓	In vitro ✓/In vivo (animal) ✓/Clinical ✗
Key advantages	Indirect modulation avoids viability constraints; potential for simultaneous immune and ecological effects; established safety profile for food-grade substrates.	Direct ecological competition and host pathway modulation; strain-specific targeting; mechanistic flexibility.	No viability requirements; improved stability and shelf-life; reduced infection risk in immunocompromised patients; better manufacturing control.
Key limitations	Sparse clinical validation in wound settings; lack of strain-level readouts; mechanisms often inferred rather than proven; functional benefits cannot be assumed across wound types.	Requires viable delivery at the wound surface; stability challenges with antiseptics and dressings; strain identity and dose are rarely genome-verified in trials; limited randomized controlled trial data.	Heterogeneous preparations in the literature; undefined dose–response relationships; absence of clinical trials; mechanistic claims often not linked to defined bioactive components.
Critical translational gap	Benefits in wounds remain largely extrapolated from in vitro selective modulation data; clinical evidence is limited to β-glucans in one wound type.	Only one wound type, diabetic foot ulcer, has been studied clinically; no data are available for venous leg ulcers, pressure injuries, or other chronic wound populations.	No validated clinical use case; the field requires standardized composition and potency characterization before clinical trial design.

## Data Availability

All data supporting the findings of this study are available within the paper.
